# The Innate Immunity Adaptor SARM Translocates to the Nucleus to Stabilize Lamins and Prevent DNA Fragmentation in Response to Pro-Apoptotic Signaling

**DOI:** 10.1371/journal.pone.0070994

**Published:** 2013-07-29

**Authors:** Chad R. Sethman, Jacek Hawiger

**Affiliations:** 1 Department of Microbiology and Immunology, Vanderbilt University School of Medicine, Nashville, Tennessee, United States of America; 2 Department of Medicine, Division of Allergy, Pulmonary and Critical Care Medicine, Vanderbilt University School of Medicine, Nashville, Tennessee, United States of America; 3 Department of Molecular Physiology and Biophysics, Vanderbilt University School of Medicine, Nashville, Tennessee, United States of America; University of Miami, United States of America

## Abstract

Sterile alpha and armadillo-motif containing protein (SARM), a highly conserved and structurally unique member of the MyD88 family of Toll-like receptor adaptors, plays an important role in innate immunity signaling and apoptosis. Its exact mechanism of intracellular action remains unclear. Apoptosis is an ancient and ubiquitous process of programmed cell death that results in disruption of the nuclear lamina and, ultimately, dismantling of the nucleus. In addition to supporting the nuclear membrane, lamins serve important roles in chromatin organization, epigenetic regulation, transcription, nuclear transport, and mitosis. Mutations and other damage that destabilize nuclear lamins (laminopathies) underlie a number of intractable human diseases. Here, we report that SARM translocates to the nucleus of human embryonic kidney cells by using its amino-terminal Armadillo repeat region. Within the nucleus, SARM forms a previously unreported lattice akin to the nuclear lamina scaffold. Moreover, we show that SARM protects lamins from apoptotic degradation and reduces internucleosomal DNA fragmentation in response to signaling induced by the proinflammatory cytokine Tumor Necrosis Factor alpha. These findings indicate an important link between the innate immunity adaptor SARM and stabilization of nuclear lamins during inflammation-driven apoptosis in human cells.

## Introduction

Innate immunity and apoptosis are intertwined defense responses of multicellular organisms to various environmental cues. The mainstays of innate immunity are molecular pattern recognition receptors known as Toll-like receptors (TLRs) that sense invading microbes. The TLR family consists of 10 known human receptors which recognize ubiquitous pathogen-associated molecular patterns. Once activated, TLRs interact with select members of the MyD88 family of intracellular adaptor proteins. These receptor-adaptor interactions are mediated by a toll-interleukin-1 receptor (TIR) domain. The TIR domain represents a common structural unit in all members of the TLR family as well as all members of the MyD88 family of adaptor proteins [1] Through TIR-TIR receptor-adaptor and/or adaptor-adaptor interactions, intracellular pro-inflammatory signaling cascades are propagated resulting in increased expression of pro-inflammatory and pro-apoptotic genes [1]. Apoptosis can ensue as a result of these signaling cascades [1]–[3].

Apoptosis, a ubiquitous process of programmed cell death essential in both organismal development and innate immunity, is characterized by distinct changes in cellular integrity such as membrane “blebbing” and cell shrinkage, as well as progressive nuclear reorganization and dismantling. The structural integrity of the cell's nucleus is essential for coupling extracellular and intracellular signals to genome responses. The highly specialized nuclear membrane is supported by scaffolding that allows nuclei to adapt during cellular differentiation, proliferation, migration, and senescence. Lamins function as major structural elements of the nuclear scaffold (nuclear lamina) which forms a matrix beneath the nuclear membrane with extensions projecting throughout the interior of the nucleus [4].

The nuclear scaffolding is made of A, B, and C-type lamins that belong to the Type V intermediate filament proteins [5]. Although the mechano-transducing function of lamins is essential for keeping the nucleus in its proper physiologic size and shape, lamins also perform other important tasks. These include pre-mitotic deconstruction and post-mitotic reconstruction of the nuclear membrane, DNA synthesis and repair, chromatin organization, transcription, nuclear import, oxidant stress quenching, and signaling mediated by extracellular signal-regulated kinase and Janus N-terminal kinase [6]. Degradation of the nuclear lamina is also essential in the reorganization and dismantling of the nucleus during apoptosis [7].

The apoptotic deconstruction of the nucleus depends on a cascade of caspase proteases that cleave critical nuclear targets. The caspase cascade is activated when members of the tumor necrosis factor (TNF) family, such as TNFα, engage their cognate “death” receptors [8]. Once stimulated, these death receptors trigger activation of initiator caspases leading to the activation of two effector caspases, 3 and 6. Caspase 3 cleaves both the enzyme involved in DNA repair, poly (ADP-ribose) polymerase, and an inhibitor of caspase activated DNAse, whereas caspase 6 targets nuclear lamins for degradation [9], [10]. In addition to the degradation of nuclear lamins, another distinguishing feature of nuclear deconstruction during apoptosis is the internucleosomal cleavage of chromosomal DNA. This DNA cleavage is carried out by a caspase-activated DNAse, which cleaves DNA between nucleosomes [11], [12]. Importantly, lamin degradation has been shown to be a prerequisite to DNA fragmentation [13]. Thus, the apoptosis-driven disintegration of nuclear integrity is a result of the sequential processes of lamin degradation, chromatin condensation (pyknosis) and DNA fragmentation which culminate in the orderly packaging of chromatin into apoptotic bodies, marking the end-stage of programmed cell death.

SARM is the most recent member of the MyD88 family of adaptor proteins to be identified and much of its functionality has remained obscure. Although homologues of SARM have been discovered in various arthropods and vertebrates [14]–[16], SARM is the only member of the MyD88 family that is expressed in *Carcinoscorpius rotundicauda* (horseshoe crab) and the nematode, *Caenorhabditis elegans*. In addition to possessing a TIR domain, shared by TLRs and other members of the MyD88 adaptor family, SARM also contains two centrally located sterile alpha motifs (SAM) and eight amino (NH_2_)-terminal HEAT/Armadillo (ARM) repeats. SAM domains, in general, have been shown to be involved in a wide variety of protein-protein interactions within the cell. ARM repeats are found in a growing number of intracellular proteins that function as cytoplasmic/nuclear shuttles including beta-catenin and members of the importin/karyopherin alpha family [17]–[19].

Though SARM shares a TIR domain [1], [14], [20] with other members of the MyD88 family, it is the sole member bearing both ARM repeats and SAM domains. This unusual structural mosaic led us to hypothesize that SARM is designed to translocate to the nucleus using its ARM repeats. Once in the nucleus, we postulated that SARM interacts with other proteins through its two SAM domains and/or ARM repeats to modulate the nuclear apoptotic response. In the studies presented here SARM displayed a nuclear localization pattern reminiscent of the lamin scaffold in human embryonic kidney (HEK) 293 cells. This nuclear trafficking of human SARM depended on the presence of its NH_2_-terminal ARM repeats. Furthermore, our results indicate that human SARM exerts a protective effect on nuclear lamins during apoptotic deconstruction induced by the proinflammatory agonist TNFα. These data provide compelling evidence for a novel anti-apoptotic mechanism vested in nuclear lamina stabilization by the innate immunity adaptor SARM in human cells.

## Results

### SARM is Translocated to the Nucleus Where it Forms a Nuclear Lamina-Like Lattice

Our studies of subcellular localization of SARM in human cells were based on its prevailing tissue-selective expression in human kidney and liver [14].

**Figure 1:**
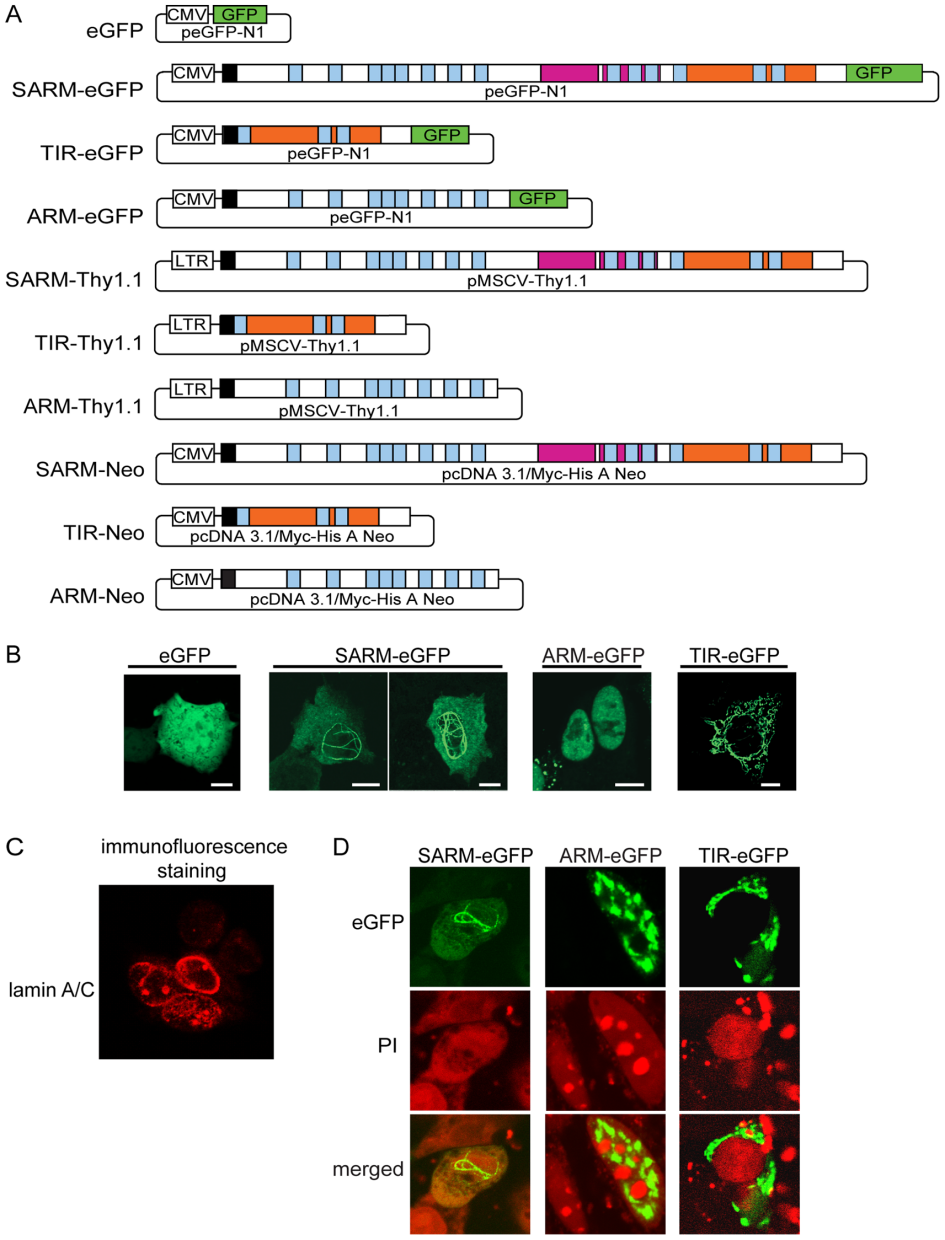
SARM constructs and their nuclear transport and localization patterns. **A.** Design of SARM constructs used in this study. SARM's modular structure is comprised of ARM repeats (blue), a SAM domain (pink) and a TIR domain (orange). All SARM constructs contain an NH_2_-terminal AU1 tag (black) to facilitate immunoblotting of expressed proteins. CMV or LTR promoters are indicated. **B.** Fluorescent confocal images showing the localization patterns of transfected eGFP and eGFP fusion proteins in HEK 293 cells. Images were taken 24 h following transient transfection. White scale bars  = 10 µm. **C.** Fluorescent confocal image of HEK293 cells fixed and permeabilized before staining with an antibody recognizing lamin A/C, visualized with a red fluorescent secondary antibody (see Materials and Methods for details). **D.** Propidium iodide (PI) counterstaining confirms nuclear localization of SARM-eGFP and ARM-eGFP and cytoplasmic localization of TIR-eGFP. Only full-length SARM-eGFP forms a lattice in the nucleus. Results shown are representative of three independent experiments.

We designed gene fusion constructs of SARM and its domains for this study as shown in Figure 1A. The full-length constructs include the complete *sarm* gene-coding sequence [14]. To identify intracellular localization patterns of full-length SARM and its domains, a fluorescent carboxy (COOH)-terminal eGFP tag was employed for cytoplasmic/nuclear tracking. An AU1 tag was added at the NH_2_ terminus to facilitate immunoblot analyses of expressed proteins in cellular extracts. Expression of eGFP alone was used as a control. Twenty-four hours after transfection to human embryonic kidney (HEK 293) cells, SARM-eGFP constitutively localized within the nucleus, forming thread-like structures looped throughout the nuclear interior (Fig. 1*B*). This pattern bears a striking resemblance to that displayed by the nuclear scaffold lamins, as depicted in published reports [21], [22]. We verified this well-known pattern of nuclear lamins by immunofluorescence staining for lamins A/C in HEK293 cells (Fig. 1*C*). NH_2_-terminal ARM-eGFP was also observed in the nucleus, but did not form the thread-like lattice seen with full-length SARM, suggesting that the protein-protein interaction domain SAM, absent in the nuclear-translocated ARM-eGFP fusion protein, may contribute to the nuclear lattice pattern. In contrast, COOH-terminal TIR-eGFP was not observed in the nucleus (Fig. 1*B*). Nuclear counterstaining of fixed cells with propidium iodide confirmed nuclear localization of the expressed SARM-eGFP and ARM-eGFP proteins (Fig. 1*D*). To assess the potential impact of attached tags on subcellular localization of SARM, we made constructs with both tags at either end of human SARM. Varying the position of the EGFP and AU1 tags did not affect SARM subcellular localization in human cells (not shown). Previous studies, including our own with MyD88-tagged AU1, indicated that presence of an AU1 tag did not change the known function of intracellular MyD88 TIR domain [23]–[25]. Likewise, the use of eGFP to track intracellular trafficking of SARM in human cells is consistent with the common application of eGFP in studies of the subcellular localization and function of several proteins, including those involved in apoptosis [26]–[30].

**Figure 2:**
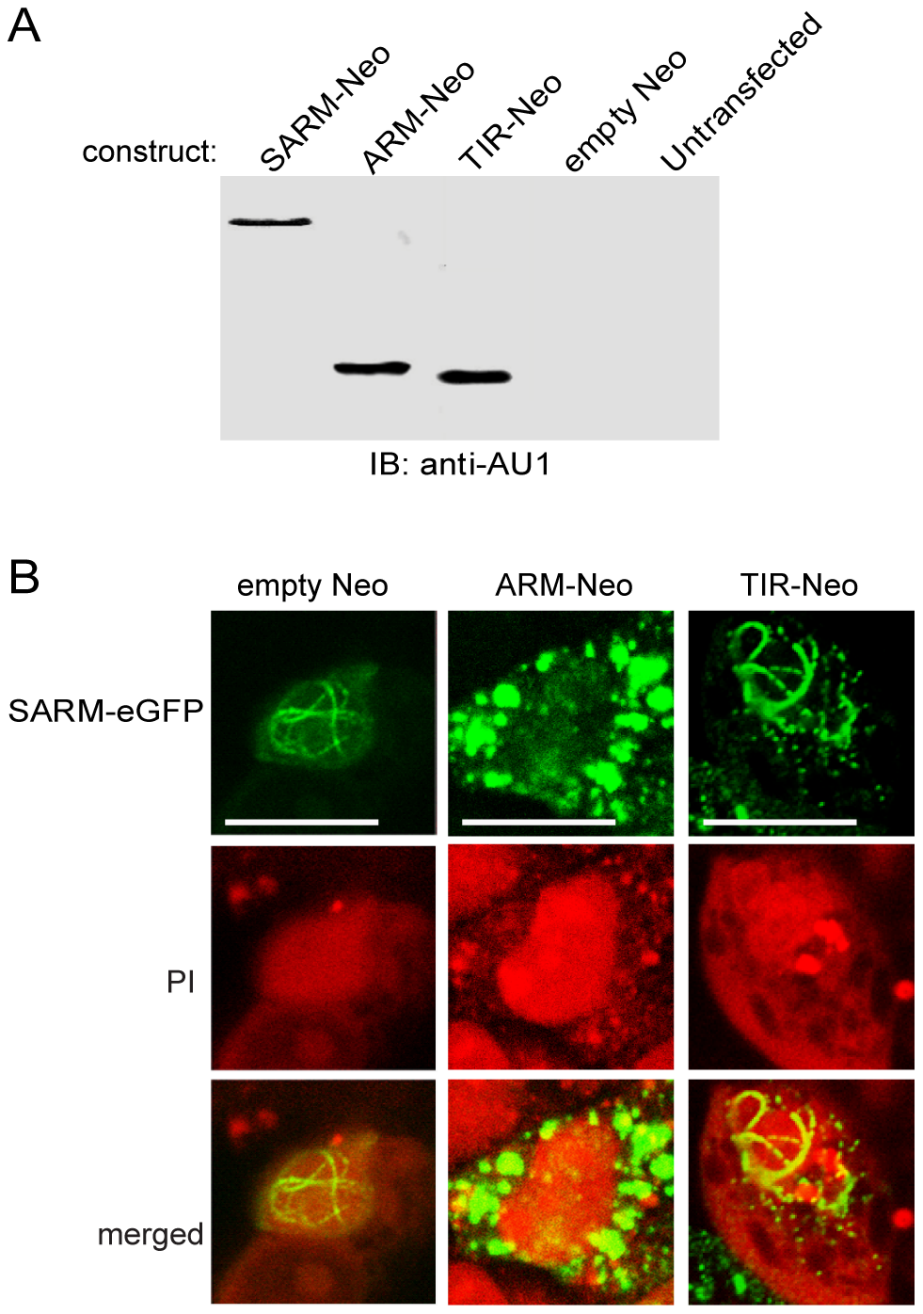
Competitive inhibition of SARM-eGFP nuclear translocation by its NH_2_-terminal ARM-repeat region. **A.** Immunoblot analysis of HEK 293 whole cell lysates. Cells were transiently transfected with the designated expression vectors. **B.** HEK 293 cells were transiently transfected with both the SARM-eGFP vector and a two-fold molar excess of either empty Neo vector or the expression vector for the non-fluorescent NH_2_-terminal ARM-repeat region (ARM-Neo) or the non-fluorescent COOH-terminal TIR domain (TIR-Neo). Fixed cells were counterstained with propidium iodide (PI) to indicate nuclei. White scale bars  = 10 µm. Results shown are representative of three independent experiments.

The nuclear transport function of the ARM-repeat domain of SARM was corroborated by a competition assay. Non-fluorescent (eGFP-free) constructs for the ARM-repeat domain (ARM-Neo), or the TIR domain (TIR-Neo) (see Fig. 1*A*) were transfected into HEK 293 cells along with the SARM-eGFP construct in a molar ratio of 2∶1, respectively. Figure 2*A* verifies expression of the non-fluorescent constructs, identified by immunoblot recognition of the NH_2_-terminal AU1 tag. Non-fluorescent ARM-Neo competitively inhibited nuclear localization of SARM-eGFP whereas TIR-Neo did not (Fig. 2*B*), indicating that nuclear translocation of SARM depends on its NH_2_-terminal ARM-repeat region. An *in silico* search of the SARM sequence (NLSdb-Database of nuclear localization signals) for a putative nuclear localization sequence (NLS) did not produce any matches.

### SARM Protects Human Cells from Apoptosis Induced by the Proinflammatory Cytokine TNFα

**Figure 3:**
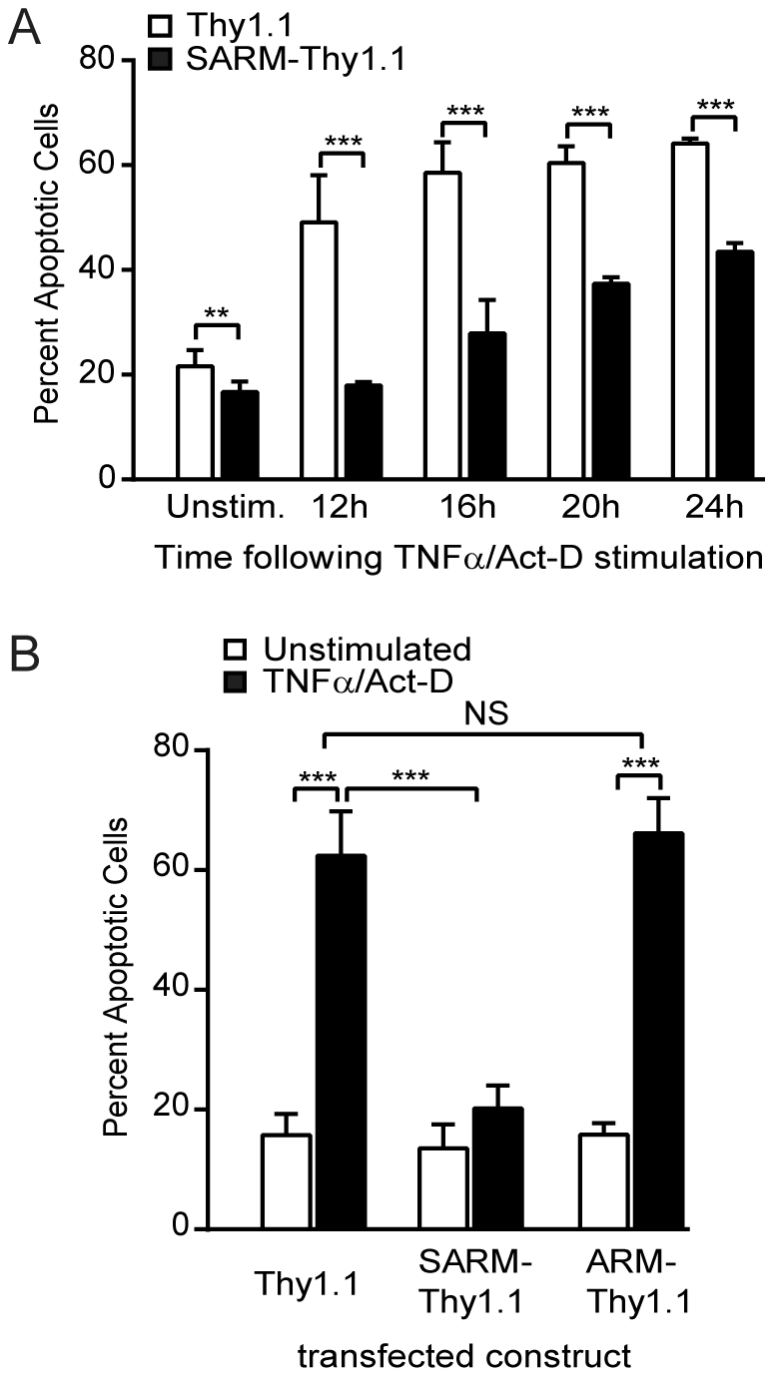
Inhibition of apoptosis by SARM. HEK 293 cells transfected with the designated constructs were stimulated 18 h after transfection with TNFα and Actinomycin-D (Act-D) for the times indicated to induce apoptosis, then stained with fluorescently-labeled anti-CD90 (Thy1.1 surface marker) and Annexin V. Apoptosis was analyzed by flow cytometry and results are expressed as the percentage of CD90-positive cells stained with Annexin V. Data are shown as the mean ± SD of three independent experiments performed in triplicate (**p<0.005; *** *p*<0.0001; NS  =  not significant). **A.** Time-dependent inhibition of apoptosis in cells expressing SARM-Thy1.1 compared to cells expressing Thy1.1 only. Cells were transfected with either SARM-Thy1.1 or Thy1.1 vectors then left unstimulated or stimulated for 12, 16, 20 or 24 h as indicated. **B.** Cells expressing SARM-Thy1.1, ARM-Thy1.1 or Thy1.1 were left unstimulated or stimulated for 14 h. Though both full-length SARM and the NH_2_-terminal ARM-repeat region translocate to the nucleus (see Fig. 1), only full-length SARM displays anti-apoptotic activity.

As noted above, SARM orthologs in the horseshoe crab and the nematode *C. elegans* were shown to protect these metazoans from death during microbial challenge [20], [31], [32]. As the observed pattern of SARM expression in the nucleus is reminiscent of the arrangement of nuclear lamina [21], [22], which is disrupted during apoptosis, we examined whether human SARM, through its nuclear localization and formation of a nuclear lamina-like lattice (see Fig. 1B), plays a role during apoptosis. The pro-inflammatory cytokine TNFα is one of the most potent and well-characterized inducers of apoptosis in human cells [8], [33], [34]. To assure that only cells expressing SARM were analyzed for apoptosis, a vector that expresses SARM along with the Thy1.1 (CD90) surface marker on the same bicistronic transcript was engineered (see Fig. 1*A*) in order to select for cells that were transiently transfected. HEK 293 cells transfected with SARM-Thy1.1 displayed significantly reduced (*p*<0.0001) apoptosis following 12 h stimulation with TNFα/actinomycin-D as compared to cells transfected with the Thy1.1 vector alone (Fig. 3*A*). In contrast, transfection of cells with the NH_2_-terminal ARM-Thy1.1 bicistronic construct was ineffective at reducing apoptosis (Fig. 3*B*). This suggests that the anti-apoptotic role of SARM is likely dependent upon its SAM domain potentially mediating stabilizing interactions with the nuclear lamina scaffold. In these experiments, SARM was not tagged with eGFP thereby adding further evidence that the prior attachment of eGFP was not favoring the nuclear localization and function of SARM. Thus, human SARM, following its ARM motif-dependent translocation to the nucleus, protects HEK 293 cells from apoptosis induced by the proinflammatory cytokine TNFα.

### Human SARM Shields Nuclear Lamins from the Degradative Function of Caspase 6 and Prevents Internucleosomal Fragmentation of DNA

**Figure 4:**
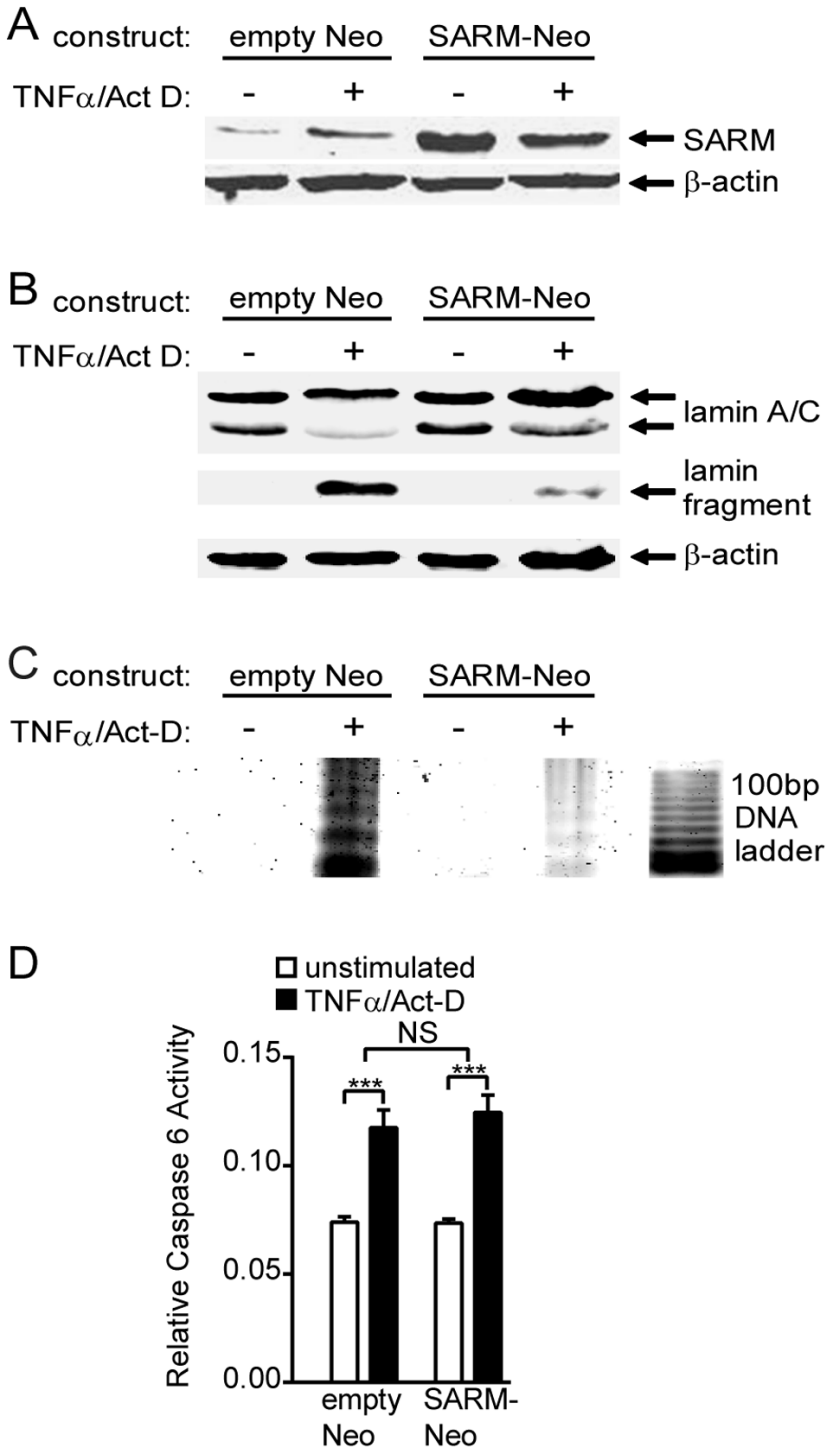
SARM inhibits lamin degradation and internucleosomal DNA fragmentation but not caspase 6 activity. HEK 293 cells stably transfected with SARM-Neo or empty Neo vector were stimulated with TNF-α and Actinomycin-D (Act-D) for the times indicated to induce apoptosis, then analyzed for lamin degradation, DNA fragmentation and caspase 6 activity. Gel segments shown in each panel represent sections of a single gel. In A and B, β-actin is shown as a loading control. **A.** Transfected cells were left unstimulated or stimulated for 14 h. Nuclear extracts were prepared and analyzed by immunoblotting with anti-SARM to confirm SARM expression. **B.** Immunoblot analysis of nuclear extracts shows a reduction of the lamin cleavage fragment in SARM-expressing cells after 14 h stimulation. **C.** Intrachromosomal DNA fragmentation (200 base pair-spaced “ladder” banding pattern) is almost completely inhibited in SARM-expressing cells after 14 h stimulation. Equal amounts of total cell DNA were loaded in each well of an agarose gel. **D.** Caspase 6 activity is not affected by SARM expression in cells stimulated for 14 h. Results shown in A–C are representative of three independent experiments. Data in D are shown as the mean + SD of three independent experiments performed in triplicate (*** *p*<0.0001; NS  =  not significant).

Activated caspase 6 (“lamin protease”) executes the specific cleavage of lamins A/C into smaller fragments as an essential step in the orderly breakdown of the nucleus during apoptosis [10], [35], [36]. To study degradation of lamins A/C, stable transfectants of HEK 293 cells were established using a full-length SARM expression vector containing the neomycin resistance gene but not eGFP (SARM-Neo; see Figs. 1*A* and 4*A*). Intact lamins A/C as well as a major caspase-dependent lamin cleavage fragment were detected in cell lysates by immunoblot analysis with a primary lamin A/C-specific antibody. Human SARM-transfected cells demonstrated a substantial reduction of the lamin cleavage fragment compared to control cells transfected with empty vector after a 14 h treatment with TNFα/actinomycin-D (Fig. 4*B*).

Apoptotic degradation of lamins A/C precedes internucleosomal fragmentation of chromosomal DNA, a distinct indicator of late-stage apoptosis [13]. A unique pattern of DNA fragmentation occurs as caspase-activated DNAse known also as DFF40 cleaves DNA at the junction of stacked nucleosomes resulting in the characteristic 180–200 base pair ladder effect seen upon agarose gel electrophoresis. Whole cell DNA was isolated from SARM- and empty vector-transfected HEK 293 cells after exposure to the same apoptotic conditions as described above. Normalized input concentrations of the DNA were then electrophoresed on an agarose gel (Fig. 4*C*). Internucleosomal DNA fragmentation was substantially reduced in cells stably transfected with human SARM as compared to cells transfected with empty vector, demonstrating that, in addition to protecting nuclear lamins from cleavage, forced expression of SARM is also able to protect DNA from internucleosomal cleavage during TNFα-induced apoptosis. This suggests that the SARM-specific inhibition of lamin A/C degradation by caspase 6 also prevents downstream DNA fragmentation. To investigate whether the lamin-protective effect of human SARM was due to inhibition of lamin protease (caspase 6) enzymatic activity, caspase 6 activity was assayed in stably transfected cells following exposure to TNFα/actinomycin-D. No significant difference in caspase 6 activity was detected between cells transfected with SARM and cells transfected with empty vector (Fig. 4*D*), indicating that SARM inhibits apoptosis by shielding lamins A/C from caspase 6 rather than by inhibiting the activation of caspase 6.

## Discussion

Taken together, these results provide a novel function for a cytoplasmic innate immunity adaptor, SARM, which protects the nucleus by stabilizing its lamin scaffolding during inflammation-driven apoptosis in human cells. In this study, SARM was translocated to the nucleus via ARM motif-dependent transport where it protected nuclear integrity from activated caspase 6 during TNFα-induced apoptosis of human cells. This protection required the protein-protein interaction domain SAM, as its absence in truncated human SARM (ARM-eGFP and ARM-Neo) was associated with loss of the nuclear lamin-associated function of full-length SARM. Furthermore, SARM prevented internucleosomal cleavage of DNA, a hallmark of apoptosis. Until now, anti-apoptotic proteins were discovered mainly through their stabilizing effect on the mitochondrial membrane [37]. In contrast, the nuclear lamina-stabilizing effect of SARM entails a new physiologic mechanism designed to protect human cells from programmed cell death.

While it is possible that the AU1 and eGFP tags employed in the SARM constructs used in our study may have had an effect on the pattern of subcellular localization observed, it is highly unlikely given the extensive use of these tags in other studies. eGFP-tagged proteins are commonly employed for studies of their subcellular localization and functional analysis, including apoptosis [26]–[30]. Immunoblotting of nuclear extracts from cells transfected with a construct that did not contain EGFP (SARM-Neo, Fig. 4A) confirm that eGFP was not needed for nuclear localization. Similarly, no anomalies in structure or function were observed with the use of an AU1 tag at the N-terminus of a related protein, MYD88 TIR [23], and analyses of epitope tags have concluded that the extremely small size of this tag (6 amino acids) makes it unlikely to exert an influence on the tagged protein [24], [25].

The current evidence regarding the role of SARM in apoptosis is evolving. SARM orthologs in *Drosophila* and horseshoe crabs have been shown to protect these organisms from death during microbial challenge [20], [31], [32]. Following West Nile virus infection, which invokes signaling through TLR pathways, SARM deficiency resulted in increased neuronal cell death [38]. In contrast, a zoonotic Bunyavirus, known as La Crosse virus, caused neuronal cell death mediated by SARM through mitochondrial damage [39]. SARM deficiency in the brains of another strain of *sarm* ^−/−^ mice resulted in decreased neuronal cell death following oxygen and glucose deprivation [40] or infection with Vesicular Stomatitis Virus [41]. Recently, a pro-apoptotic role of SARM was also demonstrated in murine T cells following influenza infection, however these cells only expressed a truncated isoform of SARM [42]. Therefore, the role of SARM in apoptosis may be bimodal, depending upon the species (mice vs humans), the type of stress stimuli and/or cell lineage (brain vs kidney).

Recent studies have also analyzed the subcellular trafficking of SARM relative to its role in apoptosis, though some discrepancies remain. An MyD88-5 (SARM)-GFP fusion protein co-localized just outside of the mitochondrial matrix in transfected monkey kidney COS-1 cells [40]. However, in HEK293T cells, transient expression of SARM-GFP localized throughout the cell and as dots in the nucleus, while a SARM-GFP construct lacking the NH_2_ terminus localized in the cytosol and was absent from the nucleus [43]. Furthermore, SARM-eGFP was shown to constitutively localize within the mitochondria of HEK 293T cells [26]. Our study extends these findings to an alternative destination of human SARM in the nucleus, documenting a potential dual function for human SARM, one in mitochondria and another in the nucleus. The discovery of a potential GRR-processing signal within the NH_2_ terminus of SARM [43] raises the possibility of post-translational processing resulting in multiple domain-specific forms of human SARM, which may independently co-localize and act within different cellular compartments. Alternative processing of SARM may also explain the inconsistency of results among recent SARM cellular localization studies. Whether or not SARM undergoes processing of this sort remains to be determined. The precise role of SARM in membrane-proximal TIR-adaptor signaling interactions is also emerging. SARM has been shown by others to inhibit proinflammatory signal transduction initiated through TLR4, one of the cognate receptors for lipopolysaccharide, a potent proinflammatory microbial virulence factor [15]. Deletion of the NH_2_-terminal segment of SARM, which consists of the ARM-repeat domains required for nuclear shuttling, as documented in this study (see Fig. 2), enhanced inhibition of TRIF- and MyD88-mediated signaling [43]. Deletion of the ARM-repeat region might therefore cause sequestration of truncated SARM in the cytosol during a stress response, enhancing the likelihood of TRIF and MyD88 inhibitory interactions.

Our findings regarding nuclear translocation of human SARM and stabilization of the nuclear lamin scaffold indicate SARM's potential for restoring balance between pro-apoptotic signals initiated through TLRs, and anti-apoptotic stabilization of lamin A/C induced by the same TLRs sensing different agonists. Our results are consistent with constitutive localization of SARM within the nucleus [43] and are, to our knowledge, the first to document the stabilizing action of SARM on nuclear lamina during TNFα–induced apoptosis. Thus, identifying SARM as an anti-apoptotic stabilizer of nuclear lamins offers a new dimension to the function of human innate immunity adaptors that comprise the MyD88 family.

As the role of inflammation in cellular senescence and aging is increasingly recognized [44], SARM expression and translocation to the nucleus induced by proinflammatory insults suggests a potential role for SARM in counteracting age-related defects of nuclear lamins. Nuclear lamins are destabilized in physiologic aging by sporadic use of the cryptic splice site in lamin A, resulting in steady generation of a truncated lamin protein referred to as progerin [45]. The findings described herein establish a novel role for SARM in linking proinflammatory signaling events of innate immunity with nuclear lamin stability in non-neuronal cells and encourage us to embark on further studies of the lamin-stabilizing action of SARM in nuclei of aging human cells.

## Materials and Methods

### Cell Culture

HEK 293 cells were obtained from the American Type Culture Collection. Cells were cultured in modified Eagle's medium (Cellgro) supplemented with 10% heat inactivated fetal bovine serum (Atlanta Biologicals), L-glutamine (2 mM), penicillin (100 units/ml), and streptomycin (100 µg/ml). Cells were maintained at 37°C in a humidified atmosphere of 5% CO_2_.

### Plasmids and SARM Constructs

Sequences representing full-length SARM (amino acids 1–690), the N-terminal ARM domain (amino acids 1–345) and the C-Terminal TIR domain (amino acids 364–690) were amplified by PCR from Human Liver QUICK-Clone cDNA (Clontech) using primers containing restriction sequences and a Kozak sequence directly followed by an AU1 tag. Two GGA repeats were inserted as a spacer between the AU1 tag and the start codon of the SARM construct. SARM constructs were cloned into the peGFP-N1 expression vector (Clontech) containing a multiple cloning site at the amino terminus of the eGFP coding region. For flow cytometric selection of transfected cells, constructs were cloned into the pMSCV-Thy1.1 expression vector which contains an ECMV-IRES element to link expression of the cloned gene with expression of a CD90 surface marker on the same polycistronic mRNA. To achieve stably transfected cell lines, constructs were cloned into the pcDNA 3.1*Myc*-HisA Neo vector (Invitrogen, Carlsbad, CA).

### Transfection of HEK 293 cells

Transient transfections of HEK 293 cells with the indicated expression constructs were performed using the *Trans*IT-LT1 transfection reagent (Mirius) according to the manufacturer's protocol. To create stably transfected cell lines, HEK 293 cells were transfected with either the SARM-Neo vector or an empty control vector also containing the neomycin resistance gene. Cells were washed and diluted in medium containing 1 mg/ml G418. After 48 h, cells were washed, subcultured, and screened for SARM expression, by immunoblotting with both anti-AU1 (Covance) and anti-SARM (United States Biological) primary antibodies. Clones were maintained in complete medium containing 1 mg/ml G418.

### Cellular Localization and Confocal Microscopy

HEK293 cells were plated in 35 mm glass-bottom microwell dishes (Mattek) 24 h prior to transfection. Cells at approximately 90% confluence were transfected with the indicated eGFP fusion constructs (see Fig. 1) with or without co-transfection with non-fluorescent SARM truncated constructs as indicated. At various time-points following transfection, adherent cells were fixed in 4% paraformaldehyde for 15 minutes. Cells were washed in PBS and, where indicated, counterstained with 1 ug/ml propidium iodide (PI) to visualize nuclei. Immunofluorescent staining of lamins in HEK 293 cells was performed on paraformaldehyde-fixed cells subsequently permeabilized with ice cold methanol:acetone (1∶1), and incubated with a rabbit polyclonal antibody recognizing lamin A/C (Abcam), followed by incubation with a highly cross-absorbed Alexa Fluor 594-labeled (red fluorescent) goat anti-rabbit secondary antibody (Invitrogen). Intracellular location of eGFP fusion proteins and lamin A/C was determined by confocal laser scanning microscopy with an inverted LSM510 META confocal microscope in the Vanderbilt Cell Imaging Core facility.

### Preparation of cytoplasmic, nuclear, and whole cell lysates

Cells were harvested by scraping and washed twice in cold PBS. Cell pellets were resuspended in cytoplasmic lysis buffer [10 mM Hepes pH 8.0, 10 mM KCl, 0.1 mM EDTA, 0.1 mM EGTA, 1x protease inhibitor cocktail (Sigma), 1 mM DTT, 0.4% NP-40] followed by incubation on ice for 10 min. Following incubation, lysate was centrifuged at 15,000×g for 10 minutes. The supernatant was separated from the nuclear pellet and saved as the cytoplasmic lysate. Nuclei were resuspended in high salt nuclear extraction buffer [20 mM HEPES pH 7.9, 400 mM NaCl, 1 mM EDTA, 1 mM EGTA, 1x mammalian protease inhibitor cocktail (Sigma P8340), 1 mM DTT]. Samples were vortexed for 15 min at 4°C, centrifuged as before, and the supernatant saved as nuclear extract. Lysates were snap frozen and stored at −80°C. Whole cell extracts were prepared as previously described [46].

### SDS-PAGE and Immunoblotting

Concentration of protein in cell lysates was quantified using a standard Bradford assay. Samples were normalized by dilution in SDS-PAGE sample buffer, boiled 5 minutes and loaded into wells of acrylamide gels. Proteins in gels were transferred to PVDF membranes during overnight transfer at 30 volts. Following transfer, membranes were blocked for 1 h at 40°C in Tris-buffered saline (TBS) with 5% non-fat dry milk (NFDM), then incubated with primary antibodies diluted in TBS with 5% NFDM overnight at 4°C with gentle rocking. Membranes were washed three times at room temperature in TBS containing 0.1% Tween 20 (TBST) before incubation with infrared dye-conjugated secondary antibodies (Rockland) diluted in TBST (1∶5000) for 1 h at room temperature. After incubation with secondary antibody, membranes were washed three times at room temperature in TBST followed by one wash in TBS. Membranes were scanned and bands were analyzed using the Odyssey Infrared Imaging System (Li-COR).

### Annexin V Apoptosis Assay

HEK 293 cells in 6-well plates were transiently transfected with either SARM-Thy1.1 or empty Thy1.1 vectors (see Fig. 1) 18 h prior to stimulation. Cells were stimulated with 5 ng/ml TNFα (Roche) and 0.5 µg/ml Actinomycin-D (Sigma) for the times indicated then stained with anti-CD90-FITC antibody (Abcam) to select Thy1.1-expressing cells and Annexin V-CY5 (BD Biosciences) to detect apoptotic cells. Analysis was performed on a FACScalibur (BD Biosciences) flow cytometer.

### Lamin Fragmentation

HEK293 cells stably transfected with either SARM-Neo or empty Neo vector (see Fig. 1) were plated in 6-well plates. Cells were stimulated with TNFα (5 ng/ml) and Actinomycin-D (0.5 µg/ml) for 14 h or left unstimulated. Nuclear extracts were prepared and normalized for total protein concentration. Immunoblotting of nuclear extracts was performed using a polyclonal anti-SARM antibody (United States Biological) recognizing the C-terminus of SARM and a polyclonal antibody to lamin A/C (Abcam) that recognizes both full-length lamin A and C (∼70 kDa) and a 28 kDa lamin cleavage fragment. Anti-β-actin (Abcam) was used as a control for protein loading.

### DNA Laddering

Nuclear extracts were prepared as described above and analyzed for DNA laddering using the Quick Apoptotic DNA Ladder Detection Kit (BioVision) according to the manufacturer's protocol.

### Caspase 6 Protease Assay

Apoptosis was induced and cells were harvested as indicated above. Caspase 6 activity was analyzed using a Caspase-6/Mch2 Protease Assay Kit (Oxford) according to the manufacturer's protocol.

### Statistical Analysis

An unpaired Student's *t* test with Welch's correction was employed to analyze differences between indicated columns in apoptosis and caspase assays. In all analyses, a *p* value less than 0.05 was considered significant.
